# Responsible Gambling Telephone Intervention to High-Risk Gamblers by a State-Owned Gambling Operator in Sweden: Study Protocol for a Study on Effectiveness, User Satisfaction, and Acceptability

**DOI:** 10.3390/ijerph17239069

**Published:** 2020-12-04

**Authors:** Anders Håkansson, Katja Franklin, Maria Dahlström, Axel Lyckberg

**Affiliations:** 1 Division of Psychiatry, Department of Clinical Sciences Lund, Faculty of Medicine, Lund University, 221 00 Lund, Sweden; 2 Region Skåne, Malmö Addiction Center, 205 02 Malmö, Sweden; 3 AB Svenska Spel, 621 80 Visby, Sweden; katja.franklin@svenskaspel.se (K.F.); maria.dahlstrom@svenskaspel.se (M.D.); axel.lyckberg@svenskaspel.se (A.L.)

**Keywords:** problem gambling, gambling disorder, behavioral addiction, motivational intervention, COVID-19

## Abstract

Gambling disorder is associated with severe financial, social, and psychological consequences, but treatment-seeking rates have been described to be low. Apart from formal treatment, motivational interventions in nontreatment-seeking high-risk gamblers have been shown to be promising. However, little is known about the effectiveness and acceptability of such motivational interventions carried out by a gambling operator as part of the company’s responsible gambling policies. Early experiences of such interventions are limited by the risk that gambling in individuals reached with the intervention may continue with a different gambling operator. The present study aims to evaluate effectiveness and user acceptability of a responsible gambling intervention continuously carried out by the Swedish state-owned gambling operator Svenska Spel Sport & Casino. This intervention for high-risk gamblers, identified either through substantial monetary losses or through a voluntary self-test by the gambler, includes a motivational telephone intervention aiming to encourage the gambler to set deposit limits, practice self-exclusion, or seek help. This protocol paper describes the two-tailed evaluation of this intervention: (1) A retrospective, register-based study of the effectiveness of the motivational intervention on gambling expenditures, deposit limits, and self-exclusions in comparison to control individuals not reached by the intervention, including all clients reached or attempted to be reached during September 2019–April 2020 (total *n* = 3626), as well as a one-to-one matched comparison of clients reached (*n* = 1404) and not reached; and (2) a prospective web survey study in individuals reached by the same ongoing telephone intervention practice from November 2020 (target *n* = 200), measuring clients’ attitudes to the intervention, perceived effects of the intervention on gambling, and their self-reported gambling on all operators after the intervention.

## 1. Introduction

Problem gambling is a public health challenge that affects many individuals within the adult general population worldwide. Previous data have shown a prevalence ranging from markedly below 1%, up to almost 6% across different studies and geographical settings [1]. Within the group of individuals with problematic gambling behaviors, a significant subgroup is affected by a gambling disorder, which is a psychiatric diagnosis categorized as one of the addictive disorders in the statistical manual of the American Psychiatric Association [2] and which is known to cause significant health hazards and a range of other consequences in affected individuals. The latter includes severe financial consequences and mental health conditions [3], as well as an increased risk of suicidal behavior [4]. Likewise, gambling disorder is known to have profound negative impacts on the loved ones of the affected person [5]. Despite difficulties of definition, up to 18% of the population has been reported to be directly or indirectly affected by the gambling disorder of index individuals [6].

There is growing evidence in favor of structured treatment for gambling disorder. Therapeutic methods that have shown beneficial effects on individuals affected with gambling disorders are cognitive-behavioral therapies [7] and different types of motivational, mainly brief, interventions [8]. Also, a number of harm-reducing interventions are self-help-oriented and based on gamblers’ own initiatives, such as various types of written self-help materials, limit-setting when gambling, and self-exclusion from gambling venues [9].

However, formal treatment-seeking in an established gambling disorder is known to be limited [10]. Also, it is known that problem gamblers’ trajectories to seeking and receiving help may be diverse, and may include strategies other than seeking formal treatment [11,12]. Based on this, there is reason to consider the gambling operator’s contact with gamblers as a window of opportunity for screening for high-risk gambling and for providing motivational or supportive interventions aiming to facilitate such treatment-seeking and to reduce gambling-related harm prior to treatment-seeking. It is of great relevance to address and intervene in hazardous gambling habits in close temporal association to gambling behavior. Here, responsible gambling practices to be adapted by gambling operators may be a promising tool in order to reach problem gamblers with motivational messages or support, helping them to reduce or quit gambling, and/or to motivate to actual treatment-seeking or other interventions, such as enrollment in voluntary self-exclusion programs.

A gambling operator’s contact with gamblers in order to help decrease their gambling is intuitively in contrast with the commercial interest of a company aiming to maximize profit. Thus, there are very few research reports that have documented such harm-reducing efforts provided by a gambling operator and that have considered the fact that such initiatives intuitively aim to decrease or discontinue the client’s gambling. Such initiatives require either a legal requirement or a voluntary responsible gambling strategy from the gambling operator. Also, such interventions by gambling operators have been called for [13]. Furthermore, given the relatively limited active uptake of self-limiting interventions by gamblers themselves [14], active responsible gambling contacts from the operator’s part may be of intuitive importance. Given the potential role conflict in gambling operators addressing clients with the aim to make clients reduce their gambling involvement, such interventions have been understudied, although governmental-owned operators may present a window of opportunity for this kind of interventions [15]. Thus, much remains to be understood about how such operator-initiated harm reduction strategies are perceived by gamblers and how effective they are, and studies addressing these issues are therefore a relatively novel part of problem gambling research.

In Sweden, the state-owned gambling operator, AB Svenska Spel (corresponding to ‘Svenska Spel Ltd.’ in English language), established a responsible gambling intervention in 2018, aiming to contact gamblers demonstrating high-risk gambling behaviors in order to help motivate, reduce, or discontinue their gambling habits. Such advice may include, for example, the advice to practice self-exclusion from gambling from the operator’s own system or in the overall national self-exclusion service, or advice to apply voluntary deposit limits and time limits within the operator’s gambling sites. This intervention project was originally referred to as ‘proactive calls’ [16] and has been inspired by a long-standing tradition within the company of conducting motivational talks to land-based casino visitors who visit frequently. The telephone intervention studied here can be referred to as a motivational intervention by telephone, reaching high-risk gamblers who are either identified by the operator’s own gambling history for the specific gamblers (high losses), or by the gambler taking a self-test (the GamTest [17]) at the operator’s site, displaying that the gambling causes problems. During the study period, a team of two to three full-time employees carried out these telephone interventions. The studied data came from the AB Svenska Spel Sport & Casino branch, which hosts sports betting, online casino slots and online table games, online poker, and online bingo. The remaining branches, which were not studied here, include AB Svenska Spel ‘Tur’ (‘luck’) offering number games and lotteries, and Casino Cosmopol, the independent subcompany responsible of the state-owned land-based casinos. During the study period, the aim was to call all self-testers displaying gambling problems (reaching the ‘red’ level in GamTest [17]) and as many high-loss gamblers as possible. The exact definitions and thresholds of the high-loss group were altered over the time of data collection and differentiated with lower thresholds for young gamblers. In an internal post-hoc analysis from Svenska Spel, the average monthly loss of clients selected for the intervention due to high losses was SEK 9202 (Swedish krona, around Euro 900) for clients aged 26 years and above, compared to SEK 1709 (around Euro 170) for clients aged 18 to 25 years.

As stated above, the scientific literature on the feasibility and effect of such operator-initiated harm reduction interventions is scarce. One previous research project clearly demonstrated promising effects from a telephone intervention of the Norwegian state-owned gambling operator in gamblers with hazardous gambling habits. In a randomized study of strategies to contact gamblers with high losses [18], which, in anonymized subjects, followed the gambling operator’s own registers, the post-intervention reduction in theoretical gambling loss during 12 months was significantly larger after receiving a telephone intervention compared to a postal letter intervention and compared to a nonintervention control group [19]. Also, a sub-study from the project demonstrated that, within the database of the company assessed, an effect was seen on lottery gamblers even from the postal letter intervention, although, for potentially more addictive gambling types, such as casino gambling and electronic gambling machines, the intervention by telephone was more effective [20]. In this study, however, individuals could not be followed with self-report data, and therefore, data were not able to describe gambling on other gambling operators apart from the one responsible for the intervention. To the best of the authors’ knowledge, no other projects have studied the potential effects of a direct interventional contact provided by a gambling operator.

Thus, studies on the effects of responsible gambling intervention are needed. Indeed, more research is required regarding the specific intervention of direct contact with high-risk gamblers. Clearly, the effects on gambling and gambling-related outcome of such an intervention is of uttermost relevance. Moreover, it is important to bear in mind that many individuals with high-risk gambling behaviors are likely not to be reached by a motivational intervention requiring a telephone contact, either because of difficulties of answering the telephone in everyday life or because financial problems caused by gambling may limit individuals’ access to the telephone or may make them more prone to use prepaid telephone services and change numbers. However, beyond the studies on actual feasibility and effects, there is also a need for further studies addressing the acceptability and feasibility of such interventions. This includes the need to address the fear of possible negative side effects of such harm-reducing interventions, although research evidence supporting such fears is sparse. While such reactions do not appear to be common in responsible gambling practices, they cannot be excluded and may apply to problem gamblers to a larger extent than nonproblem gamblers [21]. Researchers have highlighted the fact that harm-reducing interventions by gambling operators may be perceived as negative and may be difficult for gamblers to separate from a commercial intent of keeping clients who keep losing money while gambling, referred to as the ‘business vs care paradox’ [15]. While such a risk could potentially be perceived differently by clients of a noncommercial, public, state-owned gambling operator, the perception of such interventions may still be influenced by the gamblers’ impression of the overall gambling market, as the majority of operators are privately owned and commercial. Given the relative paucity of empirical data in the area, such research is still needed.

One further research gap arises from the fact that direct advising contact by a gambling operator has only been evaluated with respect to data from the own operator. In addition, previous data have been based on measured gambling variables by the operator and not based on self-tests by gamblers [19,20]. Self-report data on the effects of this intervention, including on the gambler’s gambling on other operators, may provide helpful information about the acceptability of the method. This calls for further research in the area, including both subjective and objective measures of gambling outcomes and attitudes to the intervention.

For the reasons cited above, the aims of the present study are to study (1) the objective effects of the telephone intervention, with respect to gambling patterns, the uptake of responsible gambling tools, and self-exclusion 4 weeks after the intervention in comparison to the period 8 weeks prior to it, and to compare individuals reached with the intervention to those who were either not reached or who refused the call; (2) the subjective effects of the intervention on gamblers’ attitudes to gambling and their self-reported change in gambling on the same and other operators; and (3) the subjective experience of the telephone intervention among the gamblers. Further, during the study period to be included (see below), the COVID-19 pandemic affected gambling markets worldwide, mainly through the reduction of sports events during the earliest phases of the pandemic. The COVID-19 pandemic may have also had other consequences on gambling-related behavior and gambling problems. For example, the types of gambling reported in survey studies in the present setting have been subject to at least some changes, most clearly involving a likely decrease in the opportunities for sports betting during the spring of 2020 [22,23,24]. While this does not necessarily affect the uptake or effect of responsible gambling tools, the present study also includes the additional aim of (4) assessing whether the objective effects addressed above were altered in any way during the COVID-19 pandemic in Sweden compared to previous parts of the study period.

## 2. Materials and Methods

### 2.1. Setting

AB Svenska Spel is a gambling operator owned entirely by the Swedish state. Historically, in accordance with a previous gambling legislation in Sweden, AB Svenska Spel played the role of an overall gambling monopoly for a broad range of gambling types and as one of the operators in an oligopoly situation for other gambling types (along with one large company offering horse race betting and a number of lotteries, whereas online casino gambling was not allowed). Following the broad introduction of online gambling and a large influx of overseas gambling operators offering mainly online casinos and sports betting online to Swedish customers [25], the Swedish gambling market was substantially reshaped. A novel legislation has allowed online casinos and other online-based gambling operators to operate from within the Swedish market under regulations by a license system since 1 January 2019. The gambling license requires a number of responsible gambling practices, including the full adherence to a novel Swedish national self-exclusion system, where an individual can electronically self-exclude from all online-based and land-based gambling operators (except a limited number of venues offering the limited-deposit ‘restaurant casinos’ in restaurants and similar establishments, and gambling types outside the system). The Swedish license gambling market involves a large number of operators in a commercialized market of mainly online casinos and online and land-based sports or horse betting, a smaller number of operators offering restaurant-based casinos with limited deposits, a smaller number of lotteries based mainly on charity organizations and other organizations, monopoly-based state-owned full ‘international’ casinos in major cities of the country, and a monopoly-based system of land-based electronic gambling machines. AB Svenska Spel has a monopoly role in the major casinos of three (previously four) major cities and state-owned land-based electronic gambling machine markets, as well as a commercial role in land-based and online lotteries and number games, land-based and online sports betting, and online casino and similar chance-based games. The legislation postulates that gambling operators need to identify and intervene in problem gambling. While such interventions were in use prior to this legislation by the state-owned operator (AB Svenska Spel), there is not hitherto a full picture of how this type of harm-reducing efforts may be adopted by different operators.

AB Svenska Spel Sport & Casino offers a range of screening or self-help tools, referred to as responsible gambling measures. In addition to the motivational telephone intervention described here [16], these include other motivational and responsible gambling tools provided by this operator, for example, the possibility for gamblers to view an overview of their gambling expenditures within the company; to opt for a voluntary self-test of type GamTest; to be nudged with responsible gambling messages if gambling risk increase; to not be sent direct commercial advertisements if indicating high-risk gambling behaviors or attitudes; the possibility to self-exclude for 24 h for all games or 3, 6, 12, or 36 months for all or any game family; having to pre-commit to deposit limits of a chosen size; and having the possibility to use voluntary time and loss limits [26,27,28].

Treatment-seeking patients in Sweden overwhelmingly report online casino and online sports betting as the most common gambling types [29]. Recent years have seen a likely increase in problem gambling in women, typically in online casino gambling [30].

The treatment system, with respect to problem gambling, has been clearly limited, with unclear and often nonexistent ways for problem gamblers to access treatment [31] with relatively rare exceptions of specific facilities [29]. Since January 2018, however, the treatment of gambling disorder and problem gambling has been fully included in legislation regulating the responsibilities of Swedish social services and healthcare, such that treatment can be applied for and should be provided by either of these bodies or by both in collaboration. While this system is still novel and may be unevenly distributed across the country, it has led to a large increase in the formal possibility of diagnosing and treating problem gambling. In parallel with the evolving treatment possibilities, a growing self-help movement provides peer support to gamblers and families [32,33].

### 2.2. Procedures, Participants, and Variables

The present project addresses the motivational telephone intervention through both a retrospective register-based study design and a prospective individual survey study design. Both sub-studies refer to the use of motivational telephone interventions, which were carried out by callers employed by the gambling operator. This motivational telephone intervention was carried out based on either an objective screening in gambling data by the operator or because the gambler has voluntarily taken a self-test indicating a problematic gambling behavior. The callers had considerable experience from customer-related services within the company and have undergone training in motivational interviewing. Parts of the staff also had other education or training in social work and addictive disorders. Callers were continuously coached by a psychologist with a continuous focus on motivational interviewing. The continuous development and learning activities for the providers of the intervention included visits to peer support organizations which meet and support problem gamblers and their families. The second and third authors of the present paper are among the group of former or current providers of the intervention.

The intervention involved a motivational, nonjudgmental telephone conversation investigating the gamblers’ views on their gambling patterns. The purpose of the call was to influence gambling behavior and help the gambler consider changing it. The conversation was based on the ‘stages of change’ model for behavioral change as suggested by Prochaska and Di Clemente [34], and its content was adapted depending on the perceived level of motivation and change of the client. The conversation resulted in a strategy based on the client’s own motivational process and ‘change talk,’ and may have included the actual setting or lowering of deposit limits, voluntary self-exclusion from separate gambling types or from the full gambling sites of AB Svenska Spel, advice about the national voluntary self-exclusion service associated with all licensed gambling operators in the country (www.spelpaus.se) [35], or advice to seek formal treatment, to call the national helpline for gambling problems, or to seek help and support with the national peer support organization. Also, in selected cases, the possibility of a referral to a therapist has been available. At the end, a brief question was asked about the gambler’s satisfaction with receiving the present type of intervention.

#### 2.2.1. Retrospective Study

The study was carried out in the subsection of Svenska Spel referred to as ‘Sport & Casino,’ offering a range of chance-based online casino games and both online and land-based betting services on a wide range of sports. The selection of individuals for the intervention calls was based on either objectively measured high-loss gambling or on a voluntary problem gambling screen carried out by the gambler indicating high-risk gambling practices. Around 80% of the individuals were called due to high losses.

Individuals who were reached and who underwent an intervention call were studied as an intervention group and compared to a control group (individuals who were called for an intervention call but who were not reached). The study addressed individuals reached between 1 September 2019 and 30 April 2020, a period of time considered to involve a relatively stable policy for the selection of clients for intervention calls (and up to the last date of accessible data when data extraction was carried out). Despite the COVID-19 pandemic, intervention calls were still carried out, but may intervention calls involved possible changes in the gambling types recently reported by the gamblers reached in the intervention. Also, the staff routines surrounding the intervention calls differed during COVID-19 in that callers worked from home due to pandemic-related confinement, leaving the working routines of the intervention possibly altered.

The following data were collected for all included individuals throughout the study period and analyzed with respect to the preintervention period (8 weeks) and the postintervention period (4 weeks), for each of the 12 weeks throughout the individual observation period, and for each of the gambling types included. Also, the potential improvements made were calculated for the group successfully reached with the intervention in comparison to individuals who were either not reached or who were reached but refused to undergo the intervention:

- Frequency of gambling (days per week)
- Any gambling (per week) on each of the gambling types included: Online poker, online casino slots, online live casino, betting, pool games, online bingo
- Total money wagered, per week (in Swedish currency, Swedish Krona, SEK, where SEK 1 corresponds to around Euro 0.09)
- Total money lost, per week (in SEK)
- Theoretical loss (money wagered × 1-payback percentage for each gambling type, respectively)
- Incidence of self-exclusion, including the extent and duration chosen by the individual
- Incidence of voluntary gambling limits set by the individual, including the extent and durations chosen
- Degree of gambling risk/problem as measured by the responsible gambling tool Playscan [36], i.e., the estimation of gambling behavior being either ‘green’ (low-risk), ‘yellow’ (moderate-risk), or ‘red’ (high-risk gambling)
- The gambler’s answer to the direct question asked at the end of the intervention call (for the intervention group) regarding the gambler’s perception of the call
- The information (when applicable and for the intervention group) about the information provided by the caller to the gambler about possibilities of setting voluntary deposit/gambling limits and receiving one’s own gambling history information, and about the national voluntary self-exclusion service www.spelpaus.se
- Gender
- Age (in full years in the interval 18–79 years and in 5-year intervals from 80 years and above, in order to secure confidentiality)
- Reason for the intervention call (high score on self-test or high monetary losses)
- Quality control question asked during the intervention call (for the intervention group and if the gambler had filled out a self-test at the Svenska Spel gambling platform) about whether the self-test had been carried out only ‘for fun’ or not. This variable was introduced in the system in early 2020 but was used as a test of validity measuring the calls made after its introduction.

#### 2.2.2. Prospective Self-Report Study

The intervention routine described above was carried out continuously. Study participants in this sub-study potentially included all individuals who have been reached with an intervention call from the onset of this prospective data collection (November 2020) and who provided informed consent to the study. These individuals were sent a mobile phone text message on the first business day 10 days after the intervention call, with an offer to enter a web link to the information about the study. The written information informs about the study aims, the overall type of questions asked, and the absolute confidentiality of the data reported in the study. At the end of the study information, the gambler was informed above how to seek treatment for problem gambling (the social services of the municipality, general practitioner within the healthcare system or through the national information web site providing information about possible treatment options for a broad range of health conditions including gambling disorder) and about how to contact the national helpline. The gambler was also offered the possibility to ask further questions about the study to the principal investigator (first author).

Patient Information Broker (PIB) and I-Mind Consulting provided the technical solution for the web survey to be filled out by study participants. When an individual entered the link sent in the telephone text message, she/he was referred to the information and consent page. Then, she/he was potentially referred to the web survey in case informed consent was provided. Survey responses were sent to PIB/I-Mind and summarized to the first author, who had no knowledge of the identity of the study participants. The technical solution of the web survey tool explicitly did not enable any tracing of study responses back to an identified individual, such that full confidentiality could be guaranteed.

The following information was collected in the survey:

- The gambler’s positive or negative perception of the present type of intervention call, asked using the following four items: ‘Feels that the gambling operator cares about me,’ ‘makes me annoyed and angry,’ ‘left me with a mainly positive experience,’ and ‘left me with a mainly negative experience’ (five-step Likert scale answers, ranging from ‘completely agree’ to ‘completely disagree’)
- The gambler’s subjective impression of whether the intervention call has changed her/his gambling, asking one question about whether it increased her/his gambling and one about whether it decreased her/his gambling (for both variables, a five-step Likert scale ranging from ‘completely agree’ to ‘completely disagree’ is used)
- The presence of debts related to gambling (yes/no) and the presence of any involvement with the Swedish enforcement agency related to debts (yes/no)
- Any money wagered on other gambling operators than AB Svenska Spel (yes/no), and if yes, any gambling on a gambling operator on which the gambler had not gambled before (yes/no)
- Any gambling on each of the following gambling types at any time during the past year prior to the intervention call and during the time passed after the call (Svenska Spel sports betting, other operator’s sports betting, any horse race betting, land-based electronic gambling machines, Svenska Spel online casino, other operator’s online casino, land-based casino, online poker, online bingo, land-based bingo, land-based so-called ‘restaurant casino’)
- Any contact taken by the gambler with a voluntary self-help association, with formal treatment, or with the national helpline (www.stodlinjen.se, a Swedish telephone or chat helpline for problem gambling)
- Occupation (working, studying, unemployed, retired, sick leave)
- Gender (male, female)
- Age (below 25 years, 25–39 years, 40–59 years, 60 years or above)

As the questions about the gambler’s experience of and attitudes toward the motivational calls require a call to have been carried out, these items could not be compared to a control group. Instead, the research aim in this part was mainly descriptive. Also, in this prospective self-report study part, the effectiveness measures of gambling pre- and postintervention will assess gambling outside of the gambling operator involved, thus recording self-report gambling data, which cannot be collected in any other way (mainly because a central national registration of all gambling records of individuals does not exist).

### 2.3. Power Calculation

For the prospective self-report study, we will aim for a target of 200 to be included, although we estimate that the recruitment of 100 individuals will be sufficient for satisfactory analyses and for conclusions to be drawn, given the mainly descriptive nature of the study data. The study is planned to continue until 200 responses have been collected, or at most, for 1 year. If data collection turns out to be too slow, the authors may decide to discontinue data collection after 6 months. In this prospective self-report study part, which will assess only individuals who successfully underwent a motivational call and who consented to the study, the study purposes are mainly descriptive, and a power calculation is therefore of limited value.

For the objective retrospective study, expected figures of improvement are difficult to estimate from the available literature. However, it is worth noting that the included individuals represent a total population, i.e., all individuals who were reached or attempted to be reached during a full period of time who were judged to apply a relatively stable structure surrounding this novel intervention. In total, 3626 individuals were included in the study. Among them, 1440 were successfully reached, 1542 could not be reached, and 250 individuals were reached but refused to have this motivational intervention (in addition, 30 further individuals were reached but were unable, for other reasons, to have the conversation). Thus, the group reached by the intervention and the control group exceed the size of the two active conditions compared in the Norwegian study by Jonsson et al. [20], which demonstrated significant differences between the conditions. Also, for example, a predicted modest increase in self-exclusion from 15% of control individuals to 20% of intervention individuals would require two groups of 905 individuals, respectively, for a significant difference to be demonstrated on a *p* = 0.05 level. Therefore, this sample size was judged to be sufficient for statistically significant findings to be made and for robust conclusions to be drawn. Also, individuals in the intervention group were matched based on the reason for receiving the call. The individuals reached (954 called due to large losses and 234 because of a self-test) were matched (one-to-one) to the individuals who could not be reached (963 due to losses and 268 because of a self-test).

### 2.4. Statistical Methods

Calculations will be conducted in SPSS v25.0 (IBM, Armonk, NY, USA) [37]. In the prospective survey study, data will be reported mainly as descriptive statistics, with binary statistical associations between discrete variables collected within the survey. In the retrospective register studies, statistical comparisons will be made comparing the preintervention and postintervention periods and using both unadjusted models and adjusted regression models in order to identify correlates of outcome measures, such as decreases in or discontinuations of gambling activity, the uptake of self-limiting tools, and voluntary self-exclusion. In addition, in a secondary analysis of the retrospective data, individuals successfully reached by the telephone intervention will be compared in a case-control analysis to individuals who were intended for the intervention but were not reached. This case-control analysis will be carried out as a one-to-one matching of reached individuals with non-reached individuals based on the reason for receiving the call due to a high risk score from a taken self-test, and reached individuals will be matched with non-reached individuals with the same discrete intervals of monthly loss.

### 2.5. Ethical Considerations

The study (both sub-studies) was approved by the Swedish Ethics Authority (file number 2020-03281, amendment approved with file number 2020-05445). For the anonymized retrospective register study, no informed consent procedure was carried out, according to the approved ethics application. In the prospective self-report part, written information was provided to the study participants as described above, and the survey was opened and accessible only if an individual actively provided electronical informed consent to the study. The self-report sub-study did not involve any information which could be traced back to a separate individual. In the retrospective study, data was fully anonymized to the first author. For the second, third and fourth authors of the paper, access to individual data remained possible only within pre-existing routines and regulation of data management within AB Svenska Spel and according to the conditions accepted by gamblers upon registration with the company.

### 2.6. Pre-Trial Registration

Before the start of the prospective survey sub-study and before the statistical analyses of the retrospective register dataset, the study was preregistered at clinicaltrials.gov (identifier NCT04646421).

### 2.7. Publication Plan

The project aims to result in at least two publications, which will be submitted to international, English-language, peer-reviewed scientific journals, i.e., at least one study regarding the objective retrospective register sub-study and one study regarding the prospective web survey study. Given the large amount of data collection in the retrospective sub-study, it cannot be excluded that the study findings might result in more than one scientific publication.

## 3. Discussion

The present project aims to become one of the first to scientifically document the feasibility and effects of a telephone intervention carried out by a gambling operator, aiming to help individuals with risky gambling practices reduce or quit their gambling. Publications from the project will present data on effectiveness and user satisfaction with the present intervention, and relevant covariates will be discussed, including the aspects related to the potential changes to the gambling market caused by the COVID-19 pandemic at the end of the present study period, allowing for a sub-analysis of responsible gambling practices in relation to that event. One further aspect to be studied is the risk of motivational telephone calls being perceived in a more negative way, such that a caring and motivational approach from a state-owned gambling operator may need to be discussed in relation to previous literature regarding potentially annoying [21] effects or the risk of messages being understood as paradoxically increasing harm instead of the opposite [15].

## 4. Conclusions

In several aspects, we believe that the present project will contribute findings in aspects that have rarely or have never been studied before. Motivational and harm-reducing interventions carried out by gambling operators represent a research area that has been rarely addressed to date. The project includes—for the first time to the best of the authors’ knowledge—a motivational telephone intervention among individuals screening as problem gamblers in a self-test at a gambling operator. Beyond this, the present study also will assess the acceptability of the intervention, thereby providing evidence for the possibility to recommend and implement the present responsible gambling strategy to other gambling operators and to other stakeholders in the area. In addition, in contrast to the limited research experience of such interventions before, this is also the first occasion measuring such an intervention in the subgroup of online poker gamblers.
